# Genome analysis of colistin-resistant *Salmonella* isolates from human sources in Guizhou of southwestern China, 2019–2023

**DOI:** 10.3389/fmicb.2025.1498995

**Published:** 2025-01-29

**Authors:** Jingtong Wu, Yongxian Wen, Lv You, Xiaoyu Wei, Junhua Wang, Ge Zhu, Shijun Li

**Affiliations:** ^1^School of Public Health, The Key Laboratory of Environmental Pollution Monitoring and Disease Control, Ministry of Education, Guizhou Medical University, Guiyang, China; ^2^Laboratory of Bacterial Disease, Experimental Center, Guizhou Provincial Center for Disease Control and Prevention, Guiyang, China

**Keywords:** whole-genome sequence, *Salmonella*, colistin-resistant, mcr-1.1, multidrug resistance

## Abstract

**Background:**

Colistin is commonly used as a last-resort antibiotic for multidrug resistance (MDR) bacterial infections. The emergence of colistin-resistant (CL-R) *Salmonella* has become a significant public health concern. However, the prevalence of CL-R *Salmonella* in Guizhou province remains unknown. Therefore, it is necessary to monitor CL-R *Salmonella* in Guizhou and systematically elucidate their characteristics-related resistance, virulence, and molecular epidemiology to develop effective public health strategies against resistant pathogens.

**Methods:**

The CL-R *Salmonella* isolates were identified from 933 *Salmonella* isolates by antimicrobial resistance testing. To further evaluate the molecular epidemiology, the CL-R *Salmonella* isolates underwent whole-genome sequencing (WGS) analysis followed by bioinformatic analysis.

**Results:**

A total of 43 CL-R isolates (4.6%) were identified from 933 *Salmonella* isolates, of which 39 isolates being MDR (resistance to three or more classes of antimicrobials). WGS analysis revealed 34 antibiotic resistance genes (ARGs), and point mutations in the *gyrA* gene (D87Y and D87G) were identified in all 43 CL-R isolates. Only one isolate carried the mcr-1.1 gene, a known colistin resistance. All CL-R isolates were found to carry multidrug efflux pumps. Furthermore, the most common resistance gene was *aac(6′)-ly* (40 out of 43), followed by *bla_TEM-1_* (39 out of 43). The majority of CL-R isolates contained the virulence factor *spvB* and a notable diversity in other virulence factors with varied functions. Core genome multilocus sequence typing (cgMLST) revealed that 43 CL-R *Salmonella* isolates were divided into 19 cgSTs, with cgST179151 (10 out of 43) being the most prevalent. Additionally, the CL-R *Salmonella* isolates exhibited genetic similarities with human *Salmonella* isolates from Poland, Canada, and Zhejiang province. Among the 42 CL-R isolates lacking markers for CL-R, 12 single-nucleotide variations (SNVs) were observed in 24 isolates using genome-wide association study (GWAS) analysis, which was possibly associated with colistin resistance.

**Conclusion:**

This study revealed that the majority of CL-R *Salmonella* isolates in Guizhou province exhibited MDR, with complex resistance mechanisms, representing a significant public health challenge. The genetic similarities between isolates from Guizhou and other regions suggested the possibility of international transmission or shared reservoirs of resistance. These results highlighted the urgent need for enhanced surveillance and effective public health strategies to address the risks posed by these pathogens in Guizhou.

## 1 Introduction

*Salmonella* is an important foodborne zoonotic pathogen commonly associated with cases of gastroenteritis and bacteremia and is one of the leading causes of bacterial food poisoning worldwide (Threlfall, 2002). Antimicrobial resistance (AMR) in *Salmonella* is spreading in both developed and developing countries and has become one of the top 10 worldwide threats to public health (Parisi et al., 2018; Algammal et al., 2023). In recent years, MDR *Salmonella* isolates have been widely detected in many regions of China (Zhao et al., 2023; Gao et al., 2023). Furthermore, the detection rate of MDR *Salmonella* isolates in Guizhou exceeded 80%, significantly higher than the rates reported in Anhui and Zhejiang provinces of China (Wei et al., 2023; Zhang Z. et al., 2021; Wang et al., 2020). MDR *Salmonella* poses serious challenges for treatment, increases the risk of severe disease, complicates outbreak control, and highlights the urgent need for better antibiotic stewardship and alternative strategies to manage bacterial infections.

Colistin, isolated from *Paenibacillus polymyxa* in 1947, is a polycationic antibiotic active against Gram-negative bacteria (Aghapour et al., 2019). Colistin is commonly used to treat MDR bacterial infections, particularly against carbapenem-resistant Enterobacteriaceae (El-Sayed Ahmed et al., 2020; Elbediwi et al., 2019). In addition to treating clinical infections, colistin is also widely used in animal husbandry (Sun et al., 2018). The emergence of CL-R isolates means that treatment options become severely limited, potentially leading to increased morbidity and mortality. However, limited data are available on CL-R *Salmonella* in Guizhou. We investigated human *Salmonella* isolates from Guizhou province between 2019 and 2023 regarding the prevalence of CL-R *Salmonella*, its genomic characterization, resistance genes, and phylogenetic relationships. This comprehensive analysis aimed to understand the molecular epidemiology and underlying resistance mechanisms of CL-R *Salmonella*, thus providing vital information on targeted intervention strategies.

In China, the resistance of *Salmonella* to colistin has increased in recent years. The rates of colistin resistance among *Salmonella* isolates from Shaoxing City and a district in Beijing exceeded 50%, a figure significantly higher than that reported by the European Union (Zhang Q. et al., 2021; Wang et al., 2023; European Food Safety Prevention Control, 2024). Mutations in the genes of *PhoPQ* and *PmrAB* two-component systems (TCSs) result in its constitutive activation and colistin resistance (Olaitan et al., 2014). However, in 2016, a plasmid-mediated mechanism of colistin resistance was reported, which can be spread by horizontal mobilization of the colistin resistance (*mcr*) gene, posing a significant threat to public health (Liu et al., 2016). Furthermore, several other factors, including the O-antigen polymerase that existed in group D *Salmonella* serovars, and efflux pumps, have been identified as contributing to colistin resistance (Olaitan et al., 2014; Talat et al., 2024; Ricci et al., 2020). Currently, the issue of colistin resistance and its mechanisms is a global concern.

Whole-genome sequencing (WGS) is a technique for sequencing the entire genome of a species, providing complete genomic information (Bagger et al., 2024). The application of WGS technology not only helps to identify resistance genes but also reveals the evolutionary relationships between isolates through comparative genomics. This information provides a scientific basis for clinical treatment, disease prevention, and control. In this study, we analyzed the prevalence of CL-R *Salmonella* from Guizhou province and systematically elucidated their characteristics related to resistance, virulence, and molecular epidemiology based on WGS for the first time. The findings of this study significantly enhanced our understanding of the prevalence and resistance genes of CL-R *Salmonella*, facilitating the development of more effective strategies to combat this pathogen. Moreover, this study is pivotal for safeguarding public health and promoting the rational use of antibiotics, which is essential for addressing the growing issue of antibiotic resistance and providing vital information for the formulation of targeted intervention strategies.

## 2 Materials and methods

### 2.1 *Salmonella* isolates collection

A laboratory-based surveillance program for non-typhoidal Salmonella was established. All the cities, including six county-level cities (Guiyang, Zunyi, Liupanshui, Anshun, Bijie, and Tongren) and three autonomous prefectures (Qiandongnan, Qiannan, and Qianxinan) in Guizhou province, were included in this program. A total of 933 *Salmonella* were identified from stool samples of diarrhea patients in 109 general hospitals from six cities and three prefectures of Guizhou province between 1 January 2019 and 31 December 2023 (Table 1), as described previously (Wei et al., 2023). These isolates were forwarded to the laboratory of the Guizhou Provincial Center for Disease Control and Prevention (GZCDC), where isolates were recovered, and antimicrobial susceptibilities were determined. These isolates were inoculated on the *Salmonella* chromogenic medium (CHROMagar, France) and incubated at 36°C for 18–24 h. Pale purple or purple colonies were selected and inoculated onto Krebs disaccharide iron medium (KIA) and motility indole urea iron medium (MIU) at 36°C for 18–24 h (Cyclokay Biological, China). The isolates that adhered to the initial biochemistry of *Salmonella* were further identified using the MALDI-TOF MS system MS1000 (Autobio Diagnostics Co., Ltd., China). A single colony was directly smeared onto the target plate, and 1 μL of matrix solution (α-cyano-4-hydroxycinnamic acid, CHCA) was added to the mix. Then, the mixture was dried at room temperature and tested on the MALDI-TOF MS system. The identification results were classified as follows: ranging from 9.000 to 10.000 indicated “reliable species level identification”; ranging from 6.000 to 8.999 indicated “reliable genus level identification”; and ranging below 6.000 indicated “no reliable identification.”

**Table 1:** Distribution of *Salmonella* isolates from 2019 to 2023 in Guizhou province.

Region	Number of isolates (%)					
	2019 (*n* = 53)	2020 (*n* = 122)	2021 (*n* = 244)	2022 (*n* = 231)	2023 (*n* = 283)	Total (*n* = 933)
Guiyang	11 (20.8)	19 (15.6)	25 (10.2)	48 (20.8)	61 (21.6)	164 (17.6)
Zunyi	0 (0.0)	0 (0.0)	8 (3.3)	86 (37.2)	55 (19.4)	149 (16.0)
Liupanshui	3 (5.7)	6 (4.9)	46 (18.9)	9 (3.9)	26 (9.2)	90 (9.6)
Anshun	0 (0.0)	0 (0.0)	27 (11.1)	18 (7.8)	0 (0.0)	45 (4.8)
Bijie	5 (9.4)	9 (7.4)	0 (0.0)	0 (0.0)	19 (6.7)	33 (3.5)
Tongren	24 (45.3)	39 (32.0)	88 (36.1)	43 (18.6)	65 (23.0)	259 (27.8)
Qiannan	3 (5.7)	17 (13.9)	25 (10.2)	16 (6.9)	28 (9.9)	89 (9.5)
Qiandongnan	4 (7.5)	21 (17.2)	10 (4.1)	8 (3.5)	29 (10.2)	72 (7.7)
Qianxinan	3 (5.7)	11 (8.2)	15 (6.1)	3 (1.3)	0 (0.0)	32 (3.4)

### 2.2 Antibiotic susceptibility testing

The antibiotic susceptibility of *Salmonella* isolates was evaluated using the recommended micro broth dilution protocol (CLSI M100, 28th Edition) of the Clinical & Laboratory Standards Institute (CLSI, United States) (CLSI, 2023), which used the breakpoints of various antibiotics (Supplementary Table S1). A total of 17 tested antibiotics belonging to 11 categories were tested, namely penicillin (ampicillin-AM), phenicol (chloramphenicol-C), aminoglycosides (streptomycin-STS, amikacin-AN), carbapenems (ertapenem-ETP, meropenem-MEM), β*-*lactamase inhibitor (ceftazidime/avibactam-CZA, ampicillin/sulbactam-SAM), cephems (cefotaxime-CTX, ceftazidime-CAZ), sulfonamides (trimethoprim-sulfamethoxazole-SXT), tetracyclines (tetracycline-TE, tigecycline-TGC), quinolones and fluoroquinolones (nalidixic acid-NA, ciprofloxacin-CIP), macrolides (azithromycin-AZM), and lipopeptide (colistin-CL). The *Escherichia coli* ATCC 25922 was used as quality control. The CL-R isolates were defined as a MIC of ≥4 μg/mL as the breakpoint, while intermediate isolates were defined as those with 2 ≤ MIC<4 μg/mL, no sensitive breakpoint was defined. Antimicrobial resistance, including MIC values, antimicrobial resistance rates, resistance profiles, and intermediate resistance rates of CL-R *Salmonella* isolates were analyzed using WHONET 2023 software.^1^ MDR *Salmonella* was defined as resistant to at least three different classes of antimicrobial drugs, while extensively drug-resistant (XDR) *Salmonella* was defined as remaining susceptible to only one or two classes (Magiorakos et al., 2012).

### 2.3 Whole-genome sequencing (WGS)

The genomic DNA was extracted from CL-R *Salmonella* isolates using a commercial DNA Kit (QIAGEN, Germany). The quality and concentration of bacterial genomic DNA were assessed through electrophoresis on a 1% agarose gel, and analysis was performed using a Qubit 4 Fluorometer (Thermo Scientific, Waltham, USA). DNA was fragmented and tagged with adapter sequences added to the ends using a DNA Library Prep Set (MGI Tech Co., Ltd., China). The library was amplified by PCR followed by cleanup and size selection using a DNA Clean Beads Kit (MGI Tech Co., Ltd., China) to remove very short library fragments. Next, an MGIEasy Circularization Module V2.0 (MGI Tech Co., Ltd., China) was used to denature the double-stranded library into circular single-stranded DNA and then create DNA Nanoballs (DNB). Furthermore, DNB was loaded on a chip (MGISEQ-2000RS Sequencing Flow Cell) and sequenced on the BGI MGISEQ-2000 platform (MGI Tech Co., Ltd., China), using a paired-end (PE) sequencing method with reagents from the MGISEQ-2000RS high-throughput sequencing set (PE100). The raw fastq sequences were checked for quality using FastQC v0.11.7.^2^ Genome splicing was performed using SPAdes v3.0^3^ on high-quality clean reads and *de novo* genome assembly to obtain the draft genome. The quality of the spliced sequences was assessed for N50, Q30, and GC content using Quast v4.6.3.^4^

### 2.4 Bioinformatics analysis

Average nucleotide identity (ANI) values were calculated using FastANI v.1.1 to determine the degree of genomic relatedness (Jain et al., 2018). The *S. Typhimurium* isolate (LT2, Accession No. NC_003197.2) and *S. Enteritidis* isolate (P125109, Accession No. AM933172.1) were used as references. The serotype and multilocus sequence typing (MLST) of CL-R *Salmonella* isolates were predicted using the sistr_cmd tool from Galaxy online website^5^ and CGE-MLST.^6^ VFanalyzer^7^ was used to construct orthologous groups within the CL-R genomes and conduct sequence similarity searches among the datasets of the virulence factor database (VFDB) to accurately identify potential virulence factors (VFs). A resistance gene identifier (RGI) was used to predict resistance genes of CL-R genomes from nucleotide data based on homology and SNP models in the comprehensive antibiotic resistance gene database (CRAD).^8^ The results of predicting resistance genes using the RGI included information on antimicrobial resistance gene families, drug classes, and resistance mechanisms. A further analysis was conducted, comparing the resistance phenotypes with the predicted genes of the CL-R *Salmonella* isolates.

### 2.5 Phylogenetic analysis

#### 2.5.1 Core genome multilocus sequence typing (cgMLST) analysis

The gene sequences of CL-R *Salmonella* isolates from Guizhou province were screened and analyzed using cgmlstfinder v1.2.0^9^ for the core genome MLST locus dataset. The maximum likelihood (ML) tree was constructed using IQtree v2.3.5.^10^ The tree file was visualized and adjusted through the TVBOT online website^11^ (Xie et al., 2023).

#### 2.5.2 Core genome single-nucleotide polymorphism (cgSNP) traceability analysis

High-quality WGS sequences were downloaded from the public database EnteroBase^12^ that met the following requirements: *S. Enteritidis*; human host; scaffold ≤100; coverage ≥95%; and Q20 ≥ 1 Gb. The complete genome of *S. Typhimurium* isolate (LT2, Accession No. NC_003197.2) was used as the reference. SNP analysis of the core genome was performed using snippy v4.6.0^13^ to obtain the dataset of differential SNP sites between different sequences, and the phylogenetic tree was constructed using IQtree v2.3.5 (see text footnote 10). The tree file was visualized and adjusted through the TVBOT online website(see text footnote 11) (Xie et al., 2023).

### 2.6 Genome-wide association study (GWAS) analysis

For these CL-R isolates, whose resistance genes did not match the CL-R phenotype, we further used the GWAS to analyze the possible genes and alleles associated with CL-R. We selected 41 *S. Enteritidis* isolates from Guizhou as the control group, which were not resistant to colistin. The Snippy v4.6.0 (see text footnote 13) was used for variant calling with one *S. Enteritidis* isolate (P125109, Accession No. AM933172.1) as reference. Based on the variant locus, principal component analysis (PCA) was constructed using the plink v1.90b6.21 (Purcell et al., 2007). Minor allele frequency (MAF) less than 0.03 was removed. Association analysis was performed using pyseer v1.3.12 (Lees et al., 2016). A significant association was considered when the *p*-value was <0.01, and the values were automatically corrected using R (R Core Team, 2023) with “p.adjust BH” package. The results were visualized with the Manhattan plot in R using the “CMplot” package.

### 2.7 Data availability statement

The original contributions presented in the study were included in the article/Supplementary material. The CL-R isolates sequence data of this study were publicly accessible in figshare: https://doi.org/10.6084/m9.figshare.27872181. The *Salmonella* sequences of the control group (non-colistin-resistant) can be obtained in figshare: https://doi.org/10.6084/m9.figshare.27888084. The accession numbers of the downloaded isolates were also listed in figshare: https://doi.org/10.6084/m9.figshare.27887304.v1. Further inquiries can be directed to the corresponding author.

## 3 Results

### 3.1 AMR phenotype of CL-R *Salmonella* isolates

A total of 43 CL-R isolates (43 out of 933, 4.6%) were detected from 933 *Salmonella* isolates in Guizhou province. In these CL-R isolates, 29 isolates (29 out of 43) showed a MIC of 4 μg/mL, and 14 isolates (14 out of 43) showed a MIC of 8 μg/mL. Furthermore, 173 *Salmonella* isolates (173 out of 933, 18.5%) showed intermediate resistance to colistin. Among the 43 CL-R isolates, the most common resistance was to nalidixic acid (42 out of 43), followed by ampicillin (38 out of 43) and streptomycin (33 out of 43). For β*-*lactamase inhibitors, ampicillin/sulbactam resistance was present in 30 isolates (30 out of 43), while ceftazidime/avibactam was non-resistant. In addition, cefotaxime and ceftazidime were resistant in 11 and 8 isolates, respectively. Notably, the CL-R isolates were not resistant to carbapenems and tigecycline. The MIC of the CL-R isolates for various antibiotics is shown in Supplementary Table S2.

**Figure 1:**
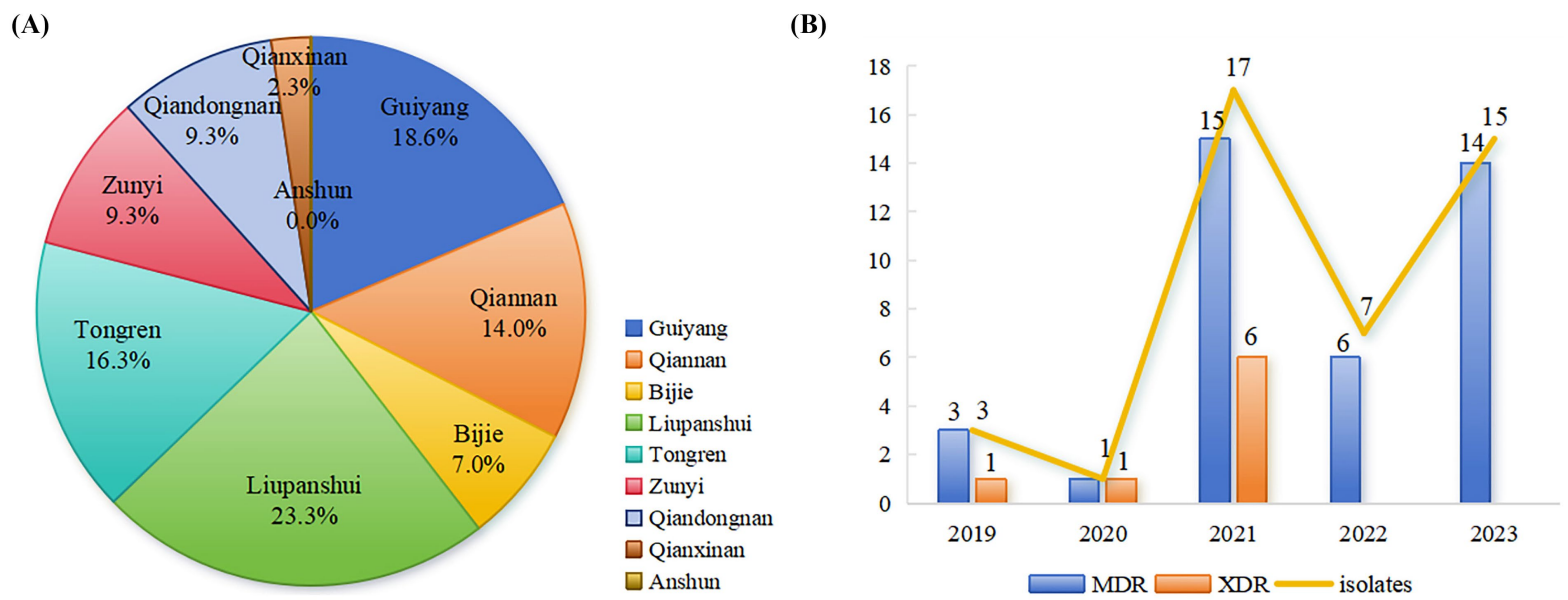
Characteristics of CL-R *Salmonella* isolated from 2019 to 2023 in Guizhou province. **(A)** Differences in detecting 43 CL-R *Salmonella* isolates from 9 cities (prefectures) are shown in different colors using pie charts. **(B)** A combination plot was used with blue bars showing the number of MDR isolates in 43 CL-R isolates and orange bars showing the XDR isolates among the MDR isolates each year. In addition, the yellow line indicates the total number of CL-R *Salmonella* isolates detected from 2019 to 2023.

All CL-R *Salmonella* isolates were distributed across eight cities (prefectures) of Guizhou province excluding Anshun, with the highest number of detections observed in Liupanshui (10 out of 43), followed by Guiyang (8 out of 43) and Tongren (7 out of 43) (Figure 1A). Among these CL-R isolates detected in 5 years, 3 isolates (3 out of 43) were detected in 2019, 1 (1 out of 43) in 2020, 17 (17 out of 43) in 2021, 7 (7 out of 43) in 2022, and 15 (15 out of 43) in 2023 (Figure 1B).

Out of the 43 CL-R *Salmonella* isolates, 39 isolates (39 out of 43) were MDR. Additionally, 8 CL-R *Salmonella* isolates (8 out of 43) defined as XDR were resistant to at least 9 antimicrobial classes, with 2 isolates (2 out of 43) resistant to 10 classes. A total of 21 unique AMR patterns were identified from 43 CL-R *Salmonella* isolates in Guizhou province (Table 2). The dominant AMR patterns were CL + NA + STS + AM + SAM (8 out of 43) and CL + TE + NA + STS + AM + SAM (7 out of 43).

**Table 2:** Unique AMR patterns of CL-R *Salmonella* in Guizhou province.

Classes	AMR pattern	Isolates (%)	Percentage (%)
2	CL + NA	3 (7.0)	9.3 (4/43)
	CL + AM	1 (2.3)	
3	CL + NA + STS	1 (2.3)	9.3 (4/43)
	CL + NA + AM	3 (7.0)	
4	CL + TE + NA + STS	1 (2.3)	7.0 (3/43)
	CL + NA + AM + SAM	1 (2.3)	
	CL + NA + STS + AM	1 (2.3)	
5	CL + NA + STS + AM + SAM	8 (18.6)	25.6 (11/43)
	CL + TE + NA + STS + AM	2 (4.7)	
	CL + CTX + CAZ + NA + AM + SAM	1 (2.3)	
6	CL + TE + NA + STS + AM + SAM	7 (16.3)	18.6 (8/43)
	SXT + CL + TE + NA + AM + SAM	1 (2.3)	
7	CL + CTX + CAZ + TE + NA + STS + AM + SAM	1 (2.3)	2.3 (1/43)
8	SXT + CL + CTX + CAZ + TE + NA + STS + AM + SAM	1 (2.3)	9.3 (4/43)
	C + SXT + CL + TE + NA + STS + AM + SAM	3 (7.0)	
9	C + SXT + CL + CTX + TE + NA + AZM + STS + AM	1 (2.3)	14.0 (6/43)
	C + SXT + CL + CTX + CAZ + TE + CIP + NA + STS + AM + SAM	2 (4.7)	
	C + SXT + CL + CTX + TE + CIP + NA + STS + AM + SAM	1 (2.3)	
	C + SXT + CL + CTX + TE + CIP + NA + AN+STS + AM + SAM	1 (2.3)	
	SXT + CL + CTX + CAZ + TE + NA + AZM + STS + AM + SAM	1 (2.3)	
10	C + SXT + CL + CTX + CAZ + TE + CIP + NA + AZM + AN + STS + AM + SAM	2 (4.7)	4.7 (2/43)

### 3.2 Genomic characterization of CL-R *Salmonella* isolates

**Figure 2:**
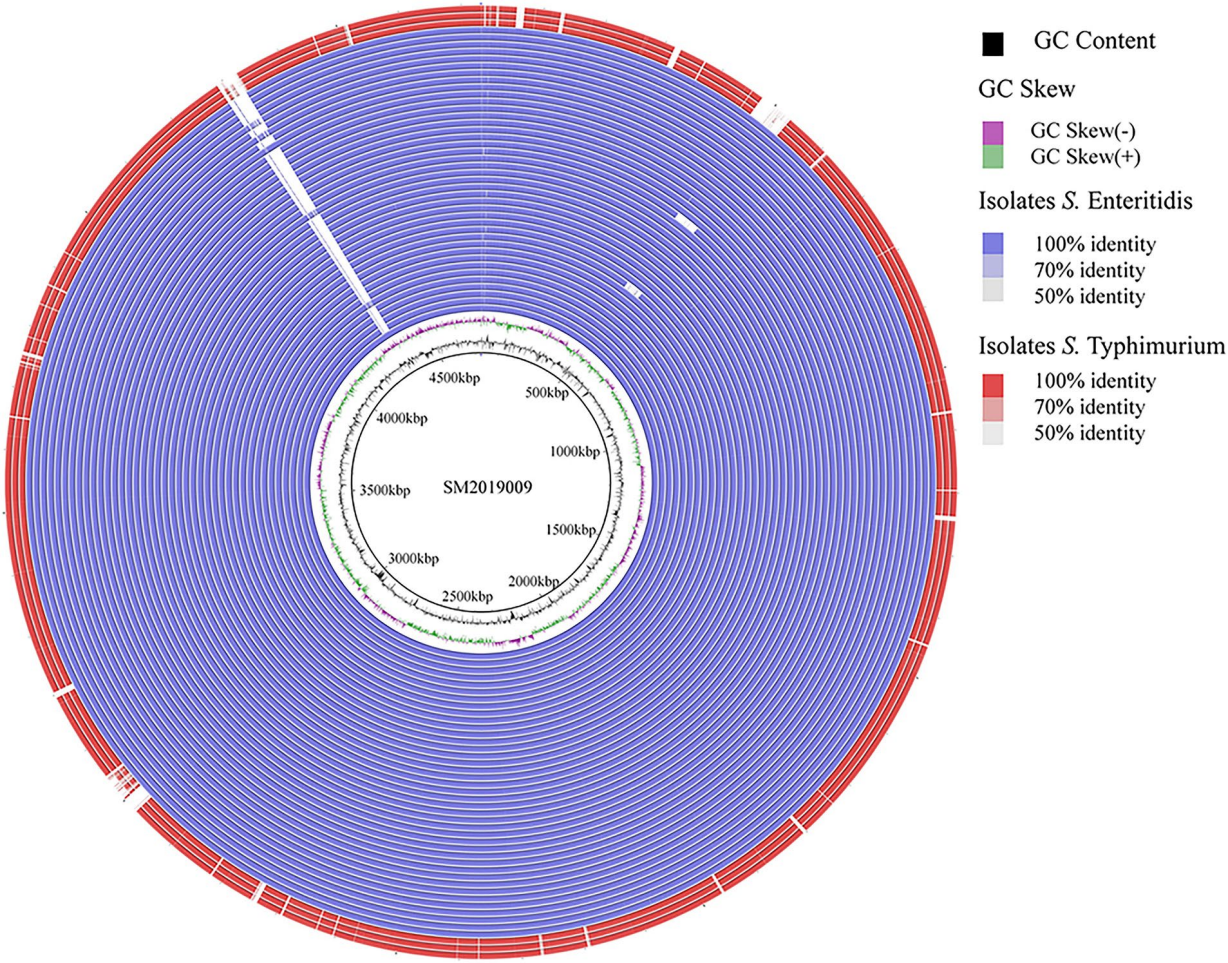
Comparative genomic map shows the differential of 43 CL-R *Salmonella* isolates from Guizhou province. We selected the genome of *S. Enteritidis* SM2019009 as the reference. The outside red circles indicate *S. Typhimurium* isolates and the other blue circles indicate *S. Enteritidis* isolates.

A total of 43 CL-R *Salmonella* isolates from Guizhou were subjected to WGS. The general information concerning the genomes and genomic characteristics of CL-R *Salmonella* isolates are presented in Supplementary Table S3. The analysis of the genomic feature showed that genome sizes of 43 CL-R *Salmonella* isolates were in the range of 4.7–5.2 Mb, with GC content and Q30 in the range of 50.61–52.35% and 91.25–97.53%, respectively. Furthermore, the values of N50 ranged from 173,706 bp to 227,815 bp for CL-R isolates. Serotype prediction showed that 40 CL-R isolates (40 out of 43) were *S. Enteritidis*, and 3 CL-R isolates (3 out of 43) were *S. Typhimurium*. Analysis of ANI found values of 40 *S. Enteritidis* genomes were >99.9%, and 3 *S. Typhimurium* genomes were also >99.9%. In addition, MLST analysis revealed that 39 (39 out of 43) isolates were typed as ST11, which were *S. Enteritidis*, and one *S. Enteritidis* isolate was untyped. Meanwhile, three *S. Typhimurium* isolates were divided into ST34 (2 out of 43) and ST19 (1 out of 43), respectively. The most frequently observed replicon type was IncFIB/IncFII (24 out of 43), followed by IncFIB/IncFII/IncX1 (13 out of 43). We selected the genome of *S. Enteritidis* SM2019009 as the reference to construct the comparative genome map of 43 CL-R *Salmonella* isolates (Figure 2). All isolates showed high sequence similarity, with only a few deletions of DNA fragments.

### 3.3 Comparison of resistance phenotype and genes in CL-R *Salmonella* isolates

**Figure 3:**
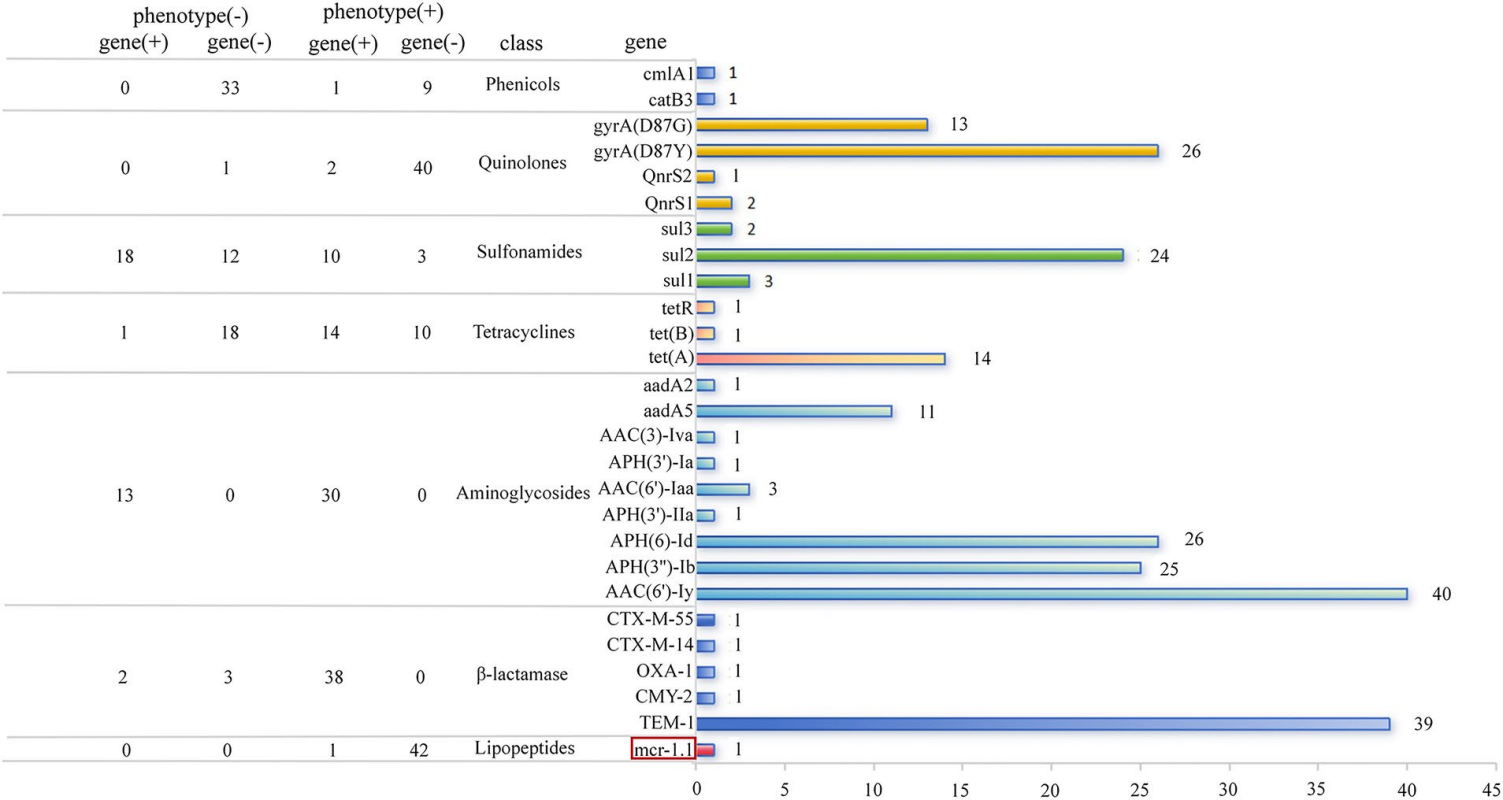
Comparison between resistance phenotype and genes carried by CL-R *Salmonella* isolates. The bar chart displays the distribution of dominant resistance genes using different colored bars, while the table compares the resistance phenotype with their corresponding resistance genes.

To understand the genetic determinants of antibiotic resistance, we searched all the sequenced genomes for genes and point mutations contributing to antibiotic resistance (Figure 3). A total of 34 ARGs and point mutations of 1 chromosomal gene (*gyrA*) were identified based on the WGS analysis, which could contribute to resistance to antibiotics belonging to 11 classes (Supplementary Table S3). We found that each genome harbored at least 11 of these ARGs. However, we found the genes conferring mcr-1 in only one (2.3%) isolate. Notably, all CL-R *Salmonella* isolates contained multiple efflux pumps, which caused resistance to multiple antimicrobial agents, such as *acrB, emrB, emrR, marA, CRP, golS, H-NS,* and *sdiA*.

ARGs that determine the resistance to aminoglycosides were contained by all isolates, among which *aac(6′)-ly, aph(6)-Id*, and *aph(3″)-Ib* were present in 40 (40 out of 43), 26 (26 out of 43), and 25 (25 out of 43) isolates, respectively. Among the genes encoding β*-*lactamase, the most prevalent one was *bla_TEM-1_*, which was contained by 39 isolates (39 out of 43). Other β-lactamase encoding genes were present in only three isolates including *bla_CTX-M-14_, bla_CTX-M-55_, bla_CMY-2_*, and *bla_OXA-1_*. The most frequently occurring sulfonamide resistance gene was *sul2*, which was encoded by 24 isolates (24 out of 43). One of the 3 types of tetracycline resistance genes, *tet(A)*, was present in 14 isolates (14 out of 43), while both *tet(B)* and *tet(R)* were found in only 1 isolate (1 out of 43). When genetic determinants contributing to the resistance against fluoroquinolones were considered, we observed that the high prevalence of the chromosomal gene mutations was observed in 39 isolates (39 out of 43), among which the point mutations were present in Asp87Tyr (D87Y) (26 out of 43) and Asp87Gly (D87G) (13 out of 43). Meanwhile, two AGRs of plasmid-mediated quinolone resistance (PMQR) genes were identified in *S. Typhimurium*, with *qnrS1* detected in 2 out of 43 isolates and *qnrS2* in 1 out of 43 isolates.

To investigate the genetic determinants behind the MDR and XDR, we examined the compositional profiles of ARGs in each genome. Among the 43 genomes containing 34 AGRs, we identified 16 combinations of distinct ARGs (Supplementary Table S3). Each combination contained an average of 19 AGRs, with the largest containing up to 26 AGRs. When drug classes targeted by these ARGs were predicted, we found that the putative MDR isolates accounted for up to 86.0% of the total, which was almost consistent with the test results of the resistant phenotype described above.

### 3.4 Presence of virulence factors for CL-R *Salmonella*

**Figure 4:**
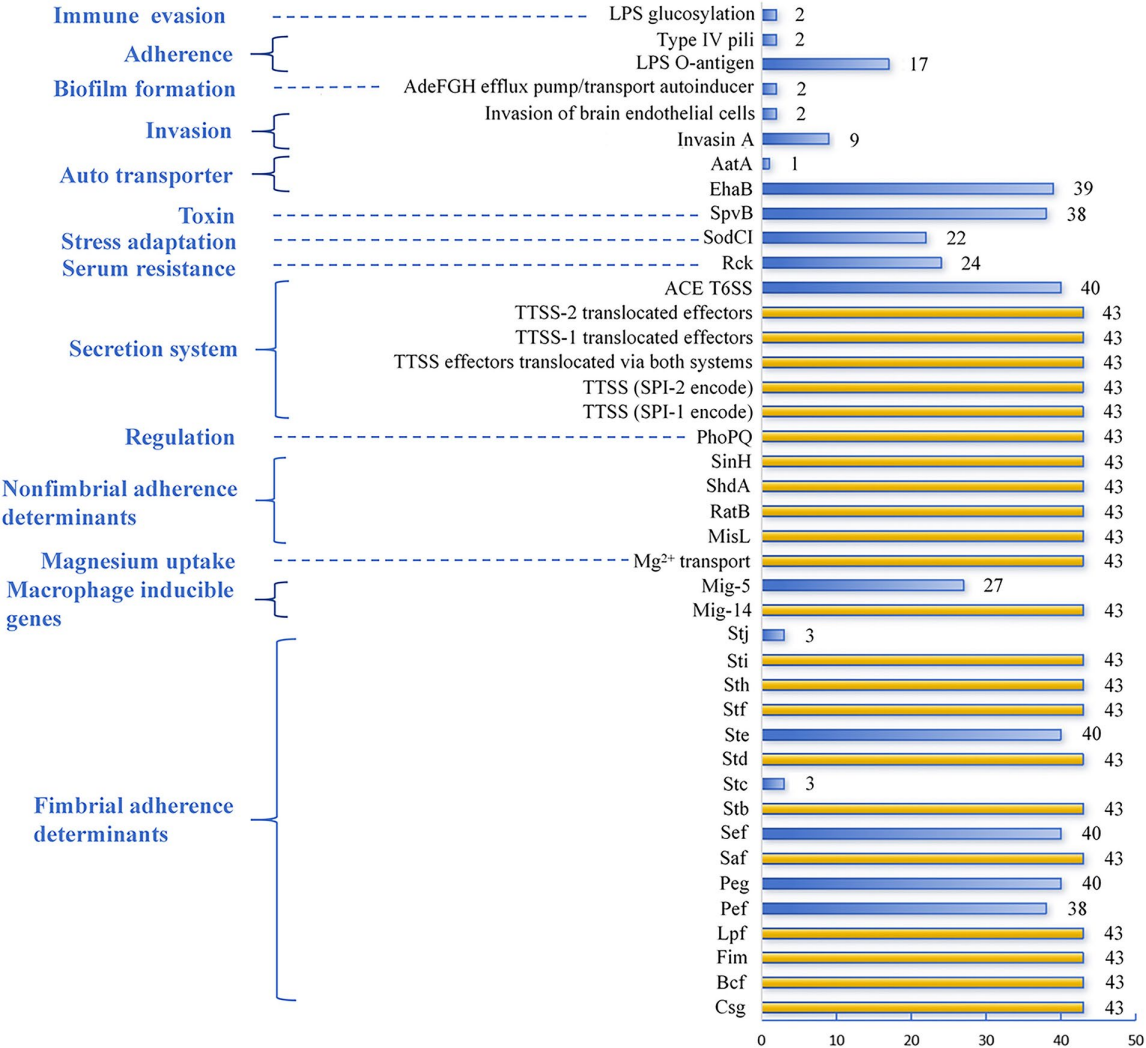
Distribution of different virulence factors among the 43 CL-R *Salmonella* isolates using a bar chart. The yellow bars represent virulence factors with a detection rate of 100%, while the blue bars represent the remaining virulence factors. The blue text on the left side of the chart indicates the virulence families of these factors.

The virulence factors generated from VFDB are based on screening WGS. As a result, 43 CL-R *Salmonella* isolates were divided into 14 groups, including immune evasion, adherence, biofilm formation, invasion, auto transporter, toxin, stress adaptation, serum resistance, secretion system, regulation, non-fimbrial adherence determinants, magnesium uptake, macrophage inducible genes, and fimbrial adherence determinants. These virulence factors are summarized in Figure 4. All isolates harbored fimbrial adherence determinants (*Sti-Sth-Stf-Std-Stb-Saf-Lpf-Fim-Bcf-Csg*), macrophage inducible genes (Mig-14), magnesium uptake (Mg^2+^ transport), non-fimbrial adherence determinants (*SinH-ShdA-RatB-MisL*), regulation (*PhoPQ*), and the secretion system (TTSS (SPI-1 encode), TTSS (SPI-2 encode), TTSS (effectors translocated via both systems), and TTSS-1 (translocated effectors), TTSS-2 translocated effectors). Meanwhile, more than 90.0% of CL-R isolates carried auto transporter (*EhaB*), toxin (*SpvB*), the secretion system (ACE T6SS), and fimbrial adherence determinants (*Ste-Sef-Peg-Pef*). In contrast, the rarest virulence factor was auto transporter (*AatA*) (1 out of 43, 2.3%), followed by immune evasion (LPS glucosylation), adherence (Type IV pili), and biofilm formation (AdeFGH efflux pump/transport autoinducer), with 4.7% (2 out of 43), respectively. Among the invasion, in addition, nine isolates carried Invasin A, the virulence factor responsible for severe *Salmonella* infections, and only two isolates carried an invasion of brain endothelial cells.

### 3.5 Phylogenetic analysis for CL-R *Salmonella*

**Figure 5:**
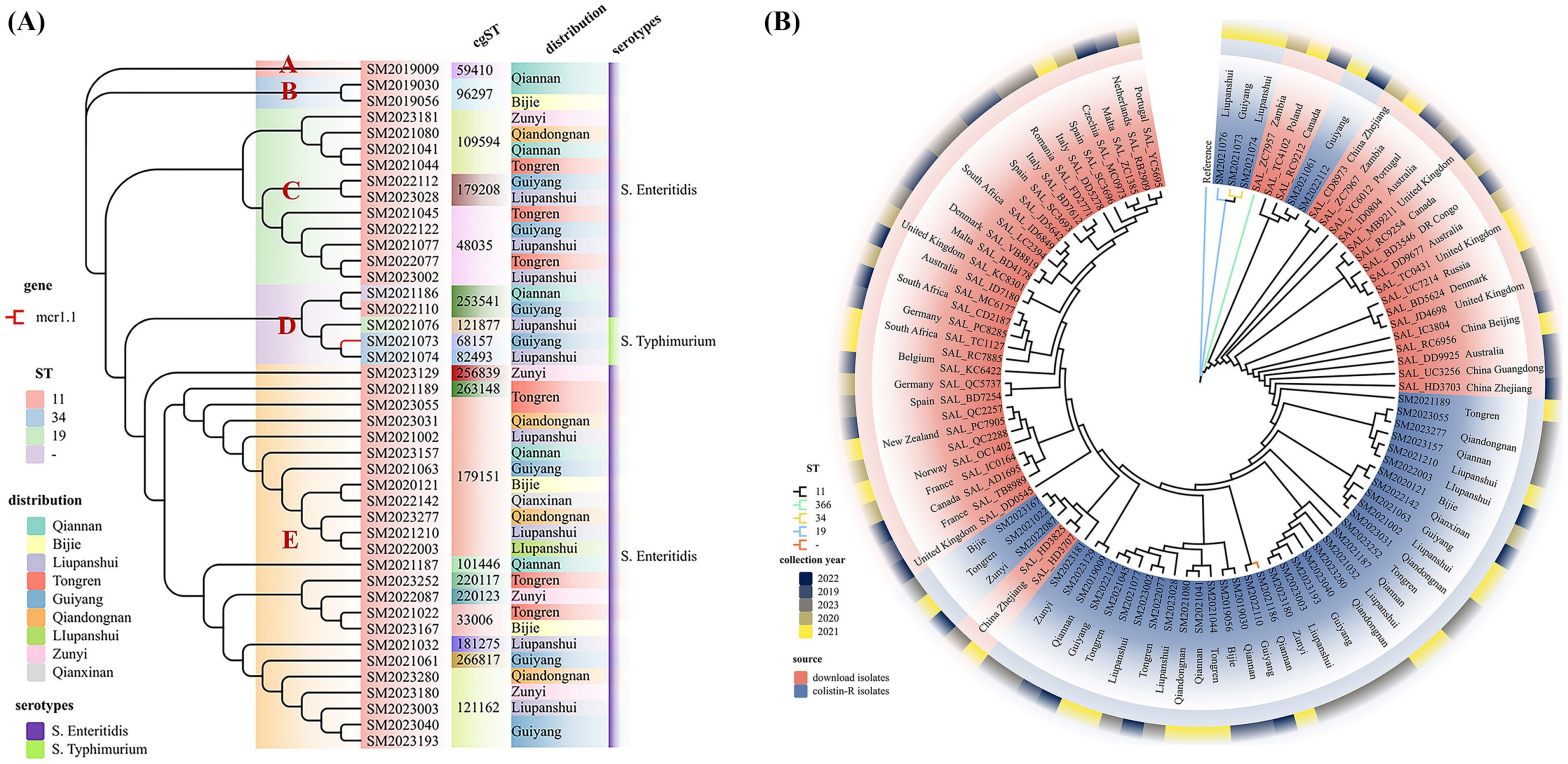
**(A)** The ML tree was constructed using cgMLST of 43 CL-R *Salmonella* isolates, which were divided into five clusters. The red branch shows the mcr-positive isolate, the red block indicates ST11, the blue block indicates ST34, the green block indicates ST19, and the purple block indicates untyped. The cgST, distribution, and serotypes of the CL-R isolates are also shown on the right of this figure. **(B)** The phylogenetic relationship of 43 CL-R *Salmonella* isolates from Guizhou province and 54 *S. Enteritidis* isolates from the NCBI database was analyzed using cgSNP. Different colored blocks on the graph indicate the source of the isolates, with blue blocks representing the CL-R isolates from our study and red blocks representing isolates from different countries obtained from the NCBI database. The MLST types of the 97 isolates are shown in different colored branches: ST11 in black, ST366 in green, ST34 in yellow, ST19 in blue, and untyped isolates in orange.

CgMLST was used to subtype 43 CL-R *Salmonella* isolates from Guizhou, which were divided into 19 cgST (Figure 5A). The dominant cgST was cgST179151, accounting for 10 isolates (10 out of 43). Notably, 40 *S. Enteritidis* isolates (40 out of 43) were classified into 16 cgSTs, and identical cgST isolates were discovered in certain cities or prefectures. The same serotype and cgST isolates were consistently present between 2020 and 2023, but no clustered outbreaks were detected. An MLST-untyped isolate, SM2021186, had a cgST type of 253,541. The phylogenetic tree was constructed based on cgMLST profiles. The analysis revealed that the 43 CL-R *Salmonella* isolates clustered according to their serotype and were grouped into 5 clusters. Twenty-four *S. Enteritidis* isolates were grouped into cluster E, while 14 isolates were in clusters A, B, and C. However, two *S. Enteritidis* isolates and three *S. Typhimurium* isolates were gathered in cluster D. Three *S. Typhimurium* isolates, SM2021076, SM2021073, and SM2021074, clustered together but were classified into different cgST types. Furthermore, only 1 of the 43 CL-R isolates, SM2021073, carried the colistin-resistant-related gene mcr-1.1 with cgST type 68157.

Based on the results of the cgMLST cluster for 43 CL-R *Salmonella* isolates, 54 *Salmonella* sequences from 22 different countries were downloaded from the website (see text footnote 12). A phylogenetic tree was constructed using cgSNP analysis of 43 CL-R *Salmonella* isolates and 54 *S. Enteritidis* isolates (Figure 5B). Within this tree, a large branch consisted of 93 *S. Enteritidis* isolates. Among them, SM2021061 and SM2022112 were found to be genetically similar to two isolates from Poland and Canada, with SNP distances ranging from 141 to 349. Similarly, SM2023181, SM2023167, SM2022087, and SM2021022 were observed to have SNP distances of 21 to 351 from 2 *S. Enteritidis* isolates, SAL_HD3825 and SAL_HD 3707, from Zhejiang province, China. Additionally, three *S. Typhimurium* isolates showed significant genetic differences from the 93 *S. Enteritidis* isolates, with SNP distances ≥30,000.

### 3.6 Genome-wide association study (GWAS) of CL-R *Salmonella* isolates

**Figure 6:**
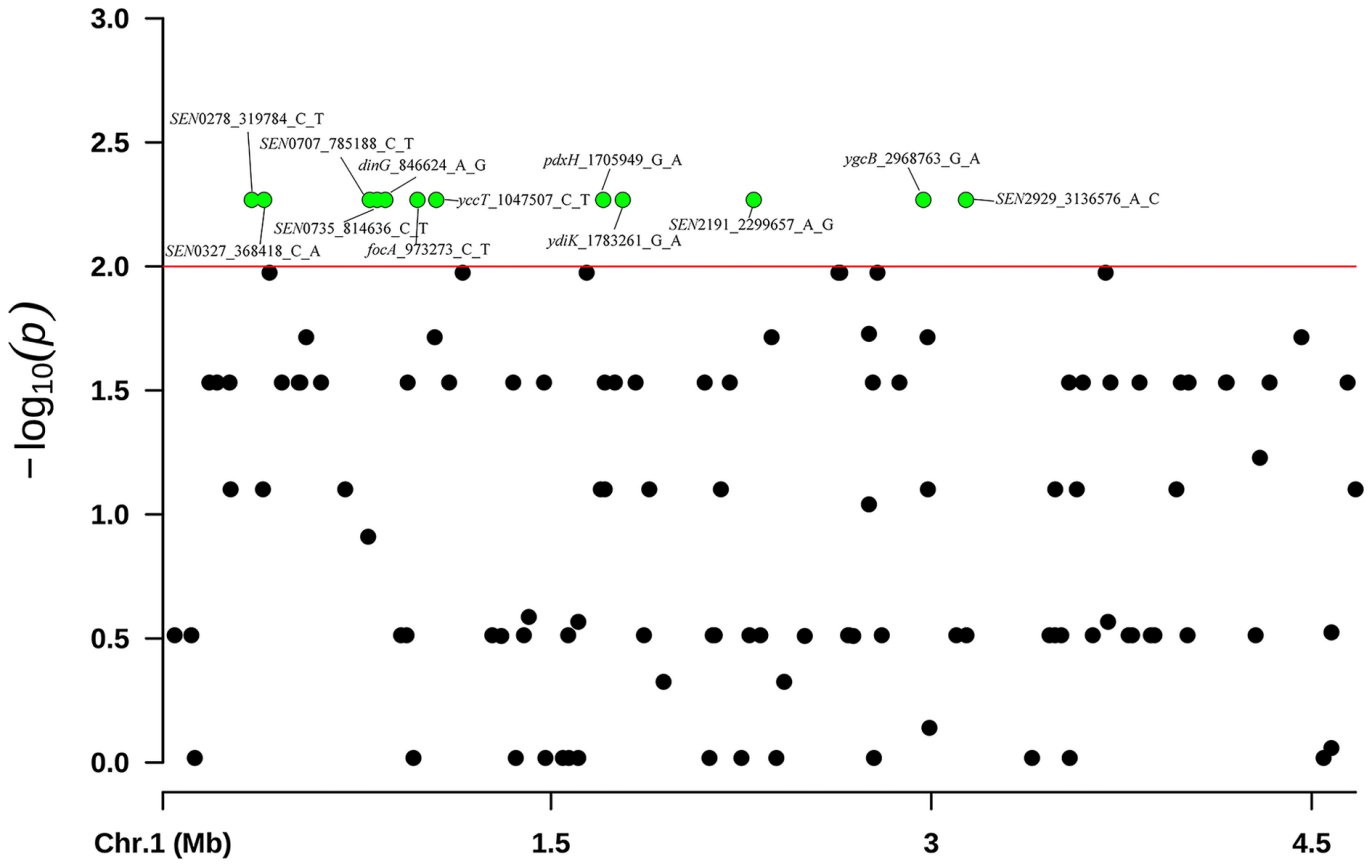
GWAS analysis was performed on 40 *S. Enteritidis* isolates of CL-R isolates and compared to 41 non-CL-R isolates. These SNVs were visualized using the Manhattan plot in R with the “CMplot” package. Green dots indicate 12 SNVs discovered in 24 *S. Enteritidis* CL-R isolates which may be a reason for these isolates being resistant to colistin. The *S. Enteritidis* isolate (P125109, Accession No. AM933172.1) was used as a reference.

Of the 43 phenotypical CL-R isolates, only 1 isolate was found to harbor the *mcr* gene. Therefore, we performed the GWAS analysis on the 40 *S. Enteritidis* isolates without the *mcr* gene to predict the potential causal SNVs associated with colistin resistance. A total of 12 variations of 12 different genes were observed in 24 isolates that were significantly associated with colistin resistance (*p* < 0.01). These genes were *dinG, focA, yccT, pdxH, ydiK, ygcB* and six unnamed genes (*SEN*0278, *SEN*0327, *SEN*0707, *SEN*0735, *SEN*2191 and *SEN*2929) (Figure 6). These 24 isolates clustered into group E in Figure 5A. The details of these genes and SNVs are provided in Supplementary Table S4.

## 4 Discussion

Salmonellosis has been one of the most common causes of bacterial gastroenteritis in Guizhou, southwestern China, over the last few decades. Surveillance data indicated that *Salmonella* infections were responsible for 92.0% of all diagnosed cases of infectious diarrhea in Guizhou province during 2018–2022, a rate significantly higher than other bacterial infections, including those caused by *E. coli* (Yang et al., 2023). In our previous study, the MDR rate of *Salmonella* was 78.5%, and the detection rate of XDR isolates accounted for 4.4% in 2013–2018 (Wei et al., 2023). The rise of MDR and XDR *Salmonella* isolates has become a serious global trend. Colistin is a last-resort antimicrobial used for treating MDR Gram-negative bacterial infections. The injudicious use of colistin in humans, animals, and the environment has led to the emergence of resistance, which has become a significant public health concern due to the potential risk of treatment failure.

In the present study, 4.6% of *Salmonella* isolates were resistant to colistin, a rate similar to findings in the European Union and animal food from China (European Food Safety Prevention Control, 2024; Guo et al., 2023). However, this rate was significantly lower than those reported by Shaoxing City and a district of Beijing (Zhang Q. et al., 2021; Wang et al., 2023). Unfortunately, the majority of CL-R isolates (39 out of 43) were MDR isolates, with eight isolates showing XDR in our study, which posed significant challenges in both clinical treatment and public health. For patients infected by CL-R *Salmonella* with MDR or XDR, the risk of treatment failure and prolonged illness is heightened due to the inability to effectively use colistin and other antibiotics. The spread of CL-R *Salmonella* can complicate outbreak control efforts. The prevalence of colistin resistance is often linked to the use of colistin in veterinary medicine, particularly in livestock, which can create reservoirs of resistance genes (Isabelle et al., 2016). This can lead to increased horizontal gene transfer among bacterial populations, further complicating the control of resistant isolates.

The discovery of *mcr* genes has obviously influenced established antibiotic therapy regimens (Liu et al., 2016). In this study, the *mcr* gene was discovered in only one CL-R *Salmonella* isolate (SM2021073), while no *mcr* genes were found in other 42 CL-R *Salmonella* isolates. Chromosomal mutations in two component systems (TCSs), *PhoPQ* and *PmrAB,* contributed to colistin resistance (Nirwan et al., 2021). Mutations within the *pmrA* and *pmrB* or *phoP* and *phoQ* genes give rise to constitutive activation of the *PmrAB* and *PhoPQ* TCSs, respectively, and lead to the upregulation of the *pmrCAB* and *pmrHFIJKLM* operons. This gives an increased synthesis of phosphoethanolamine (pEtN) and 4-amino-4-deoxy-L-arabinose (L-Ara4N) and their addition to LPS, thus reducing the efficacy of colistin and polymyxin B against isolates harboring mutations in these genes (Ricci et al., 2020). However, in our study, chromosomal mutations in TCSs were not observed in any of the CL-R *Salmonella* isolates, indicating that TCS mutations were not a factor in colistin resistance in Guizhou. Efflux pumps, which typically exhibit broad substrate specificity, are capable of recognizing and expelling a wide range of drugs. By sequestering and expelling antibiotics from the intracellular milieu to the extracellular environment, these pumps effectively reduce intracellular antibiotic concentrations, thereby impeding antibiotic access to their molecular targets (Gurvic and Zachariae, 2024). In our study, all CL-R isolates carried multiple efflux pumps. These efflux pumps may contribute to colistin resistance and MDR in Guizhou. Moreover, the serotype prediction revealed that 40 CL-R isolates (40 out of 43) were *S. Enteritidis*, belonging to group D *Salmonella* serovars in this study. Agersø et al. (2012) suggested that some serovars of *Salmonella*, namely, those belonging to group D, appeared to show a degree of intrinsic resistance to colistin. Ricci et al. (2020) mentioned that in group D *Salmonella* serovars, the O-antigen polymerase, which controlled their antigenic specificity, may induce intrinsic resistance to colistin. This may be another factor contributing to their resistance to colisin in Guizhou. Beyond the known determinants associated with colistin resistance, there may be other underlying factors contributing to the emergence of colistin resistance in *Salmonella* isolates from Guizhou province. We further conducted the GWAS analysis by mapping quantitative trait loci (QTL) to better understand the genetic structure and identify DNA variants associated with colistin resistance. This approach used various statistical methods to assess the association between genotypes and phenotypes (Goddard and Hayes, 2009). In our study, 12 SNVs were detected in 24 CL-R isolates using the GWAS analysis, which may have contributed to their resistance to colistin. It was the first time the GWAS analysis has been performed for colistin resistance, and further research is necessary to determine its relevance. Colistin resistance in Guizhou was complex, with the mcr gene, efflux pumps, O-antigen polymerase, and SNVs identified as potential contributors in Guizhou. Efflux pumps help bacteria expel colistin, while O-antigen polymerases modify cell wall structures to prevent drug binding. SNVs can alter gene functions, enhancing resistance. These mechanisms challenged healthcare providers and underscored the urgent need for new treatment strategies and better antibiotic stewardship programs. Meanwhile, researchers should work on rapid diagnostic tools to identify colistin-resistant bacteria and monitor resistance gene spread.

For other resistance genes, the majority of CL-R *Salmonella* isolates carried the *aac(6′)-ly* gene, which is one of the essential chromosomal genes responsible for modifying aminoglycoside, leading to resistance. Furthermore, among the CL-R *Salmonella* isolates from Guizhou that were resistant to quinolones and fluoroquinolones, the main mechanism was amino acid substitutions in the quinolone resistance-determining region (QRDR) of the *gyrA*, with 39 isolates (39 out of 43) showing clustering in codons 87. The results of the present study were similar to those reported by other studies in this field (Heisig, 2003). In *Salmonella*, mutations caused by amino acid substitutions in *gyrA*, including serine to phenylalanine at codon 83 (S83F), and from aspartic acid to asparagine (D87N), glycine (D87G), or tyrosine (D87Y) at codon 87, are frequently observed (Misra et al., 2016). Our study showed that Asp87Tyr (D87Y) was identified in 26 isolates and Asp87Gly (D87G) in 13 isolates. Additionally, the predominant β*-*lactamase resistance gene of CL-R isolates in Guizhou was *bla_TEM-1_*, which belongs to the extended-spectrum β*-*lactamase (ESBL) gene and hydrolyzes a wide range of antimicrobial drugs (Cheng et al., 2022). Other ESBL genes were present in our two CL-R *Salmonella* isolates, including *bla_CTX-M-14_* and *bla_CTX-M-55_*, which led to resistance to third-/fourth-generation cephalosporins. In addition to being highly resistant to β*-*lactamases, isolates harboring the ESBL gene may also be resistant to other antimicrobials, including aminoglycosides, tetracyclines, and quinolones (Kuang et al., 2018). Therefore, it is essential to strengthen dynamic surveillance of resistant-ESBL *Salmonella* isolates from food, animals, and humans to provide the basis for control and prevention efforts.

Clinical pathogenicity of *Salmonella* usually depends on the bacterial load, the infection site of the host, and the production of bacteriotoxin such as ADP ribosylating toxin protein *SpvB* (Fàbrega and Vila, 2013). Cell invasion and biofilm were two significant virulence determinants of *Salmonella* pathogenesis and host response depending on virulence factors (Fàbrega and Vila, 2013; Bronnec et al., 2016; Galvão et al., 2016; Jajere, 2019). Furthermore, flagella, plasmids, adhesion systems, *Salmonella* pathogenicity islands (SPIs), and its type III secretion systems (T3SSs) have been demonstrated as the critical virulence factors of *Salmonella* (Daigle, 2008; Sabbagh et al., 2010). The virulence genes detected in this study primarily function in toxin gene *SpvB*, type IV secretion systems (T6SSs), regulation, fimbrial and non-fimbrial adherence determinants, magnesium uptake, and macrophage inducible genes. These above virulence genes were the main pathogenic factors in all CL-R *Salmonella* isolates in Guizhou. On the other hand, the virulence genes that function in cell invasion and biofilm formation were just discovered in several isolates. These results indicated that the pathogenicity of *Salmonella* isolates differed from that observed in the previous study, warranting further investigation to understand the underlying reasons (Jajere, 2019).

CgMLST focuses on identifying slight differences in the 3,002 conserved genes of *Salmonella* to create a high-density marker system for analyzing bacterial phylogeny and population structure (Casadesús et al., 2018). In our study, only 3 STs were discovered through MLST but 19 cgSTs were identified using cgMLST in 43 CL-R *Salmonella* isolates. This result indicated that the cgMLST was more capable of detecting subtle differences between isolates compared to traditional MLST. The isolates with the same cgST persisted between 2020 and 2023 in certain cities or prefectures, and no clustered outbreaks were detected. This suggested the possibility of cross-regional transmission and a common original contaminant. The persistence of certain cgSTs over multiple years in specific geographic regions suggests that there may be environmental reservoirs or ongoing sources of contamination that require further investigation. Understanding the pathways and mechanisms of *Salmonella* transmission is crucial for developing effective intervention measures to reduce the incidence of foodborne illness. Traceability analysis showed that five CL-R *Salmonella* isolates shared genetic similarities with human-derived *Salmonella* isolates from Poland, Canada, and Zhejiang province, indicating the potential for transnational and interprovincial transmission of CL-R *Salmonella* isolates. The discovery of genetic similarities between *Salmonella* isolates from different countries and provinces highlights the importance of international collaboration and data sharing in the surveillance of infectious diseases. It is crucial to establish a comprehensive framework for monitoring and managing CL-R *Salmonella*, including healthcare, agriculture, and food safety. Future research should build on these findings by exploring the environmental and host factors that contribute to the persistence and spread of CL-R *Salmonella.* Additionally, developing new diagnostic and therapeutic approaches to combat this important foodborne pathogen is crucial.

## 5 Conclusion

This study was the first time to systematically summarize the resistance and genome of CL-R *Salmonella* isolates in Guizhou province. The findings of this study showed the severity of the MDR problem in CL-R *Salmonella* isolates in Guizhou province. The majority of isolates were *S. Enteritidis*. This is of concern since the last-resort drug colistin may not be effective for treating severe human infections with the most common *Salmonella* serotype. Meanwhile, these isolates could potentially spread across regions, provinces, and even internationally. Therefore, stricter supervision of colistin use in clinical and veterinary in Guizhou province is necessary. It is essential to continue monitoring the colistin-resistance characteristics of isolates from humans, animals, and food. Furthermore, a deeper understanding of the mechanisms underlying colistin resistance is needed to inform new strategies for treatment.

## Data Availability

The datasets presented in this study can be found in online repositories. The names of the repository/repositories and accession number(s) can be found in the article/Supplementary material.

## Footnotes

^1^ https://whonet.org/software.html

^2^ http://www.bioinformatics.babraham.ac.uk/projects/fastqc/

^3^ http://cab.spbu.ru/software/spades/

^4^ https://cab.spbu.ru/software/quast/

^5^ https://usegalaxy.eu/

^6^ https://cge.food.dtu.dk/services/MLST/

^7^ http://www.mgc.ac.cn/cgi-bin/VFs/v5/main.cgi

^8^ https://card.mcaster.ca/

^9^ https://bitbucket.org/genomicepidemiology/cgmlstfinder

^10^ http://www.iqtree.org

^11^ https://www.chiplot.online/tvbot.html

^12^ https://enterobase.warwick.ac.uk/

^13^ https://github.com/tseemann/snippy
