# Supplementary material for: Genome analysis of colistin-resistant Salmonella isolates from human sources in Guizhou of southwestern China, 2019–2023
Source: Front Microbiol. 2025 Jan 29;16:1498995. doi: 10.3389/fmicb.2025.1498995 (PMC11813891; doi:10.3389/fmicb.2025.1498995)
Supplement: Supplementary file 1 [file Table_1.DOCX]

Supplementary Material

# Supplementary Tables

Supplementary Table S1. Table The breaking point of various antibiotics (μg/ml)

| **Antibiotics** | **sensitive** | **intermediate** | **resistant** |
| --- | --- | --- | --- |
| **chloramphenicol** | ≤8 | 16 | ≥32 |
| **trimethoprim-sulfamethoxazole** | ≤2/38 | - | ≥4/76 |
| **colistin** | - | ≤2 | ≥4 |
| **ertapenem** | ≤0.5 | 1 | ≥2 |
| **meropenem** | ≤1 | 2 | ≥4 |
| **cefotaxime** | ≤1 | 2 | ≥4 |
| **ceftazidime** | ≤4 | 8 | ≥16 |
| **ceftazidime/avibactam** | 8/4 | - | ≥16/4 |
| **tetracycline** | ≤4 | 8 | ≥16 |
| **tigecycline** | ≤2 | 4 | ≥8 |
| **ciprofloxacin** | ≤0.06 | 0.12-0.5 | ≥1 |
| **nalidixic acid** | ≤16 | - | ≥32 |
| **azithromycin** | ≤16 | - | ≥32 |
| **amikacin** | ≤16 | 32 | ≥64 |
| **streptomycin** | ≤8 | 16 | ≥32 |
| **ampicillin** | ≤8 | 16 | ≥32 |
| **ampicillin/sulbactam** | ≤8/4 | 16/8 | ≥32/16 |
| - : no breakpoints for *Salmonella.* | | | |

Supplementary Table S2. The MIC values of 17 antibiotics to CL-R *Salmonella* isolates in Guizhou province.

| **Isolates(n=43)** | **C** | **SXT** | **CL** | **ETP** | **MEM** | **CTX** | **CAZ** | **CZA** | **TE** | **TGC** | **CIP** | **NA** | **AZM** | **AN** | **STS** | **AM** | **SAM** |
| --- | --- | --- | --- | --- | --- | --- | --- | --- | --- | --- | --- | --- | --- | --- | --- | --- | --- |
| **SM2019009** | 4 | 2/38 | 4 | 0.25 | 0.12 | 0.25 | 0.25 | 0.25/4 | 1 | 0.25 | 0.12 | 32 | 4 | 4 | 32 | 8 | 16/8 |
| **SM2019030** | 32 | 8/152 | 8 | 0.25 | 0.12 | 16 | 8 | 0.25/4 | 16 | 0.25 | 0.25 | 32 | 32 | 4 | 32 | 32 | 16/8 |
| **SM2019056** | 4 | 8/152 | 8 | 0.25 | 0.12 | 0.25 | 0.25 | 0.25/4 | 16 | 0.5 | 0.12 | 32 | 4 | 4 | 8 | 32 | 32/16 |
| **SM2020121** | 32 | 8/152 | 4 | 0.25 | 0.12 | 16 | 16 | 0.25/4 | 16 | 0.25 | 2 | 32 | 16 | 4 | 32 | 32 | 32/16 |
| **SM2021002** | 4 | 0.5/9.5 | 8 | 0.25 | 0.12 | 0.25 | 0.25 | 0.25/4 | 16 | 0.5 | 0.25 | 32 | 4 | 4 | 32 | 32 | 32/16 |
| **SM2021022** | 4 | 0.5/9.5 | 8 | 0.25 | 0.12 | 0.25 | 0.25 | 0.25/4 | 1 | 0.5 | 0.25 | 32 | 4 | 4 | 32 | 32 | 32/16 |
| **SM2021032** | 4 | 0.5/9.5 | 4 | 0.25 | 0.12 | 16 | 16 | 0.25/4 | 1 | 0.25 | 0.25 | 32 | 4 | 4 | 16 | 32 | 32/16 |
| **SM2021041** | 32 | 8/152 | 4 | 0.25 | 0.12 | 0.25 | 0.25 | 0.25/4 | 16 | 0.25 | 0.25 | 32 | 4 | 4 | 32 | 32 | 32/16 |
| **SM2021044** | 4 | 0.5/9.5 | 4 | 0.25 | 0.12 | 0.25 | 0.25 | 0.25/4 | 1 | 0.25 | 0.25 | 32 | 4 | 4 | 8 | 32 | 16/8 |
| **SM2021045** | 4 | 0.5/9.5 | 4 | 0.25 | 0.12 | 0.25 | 0.25 | 0.25/4 | 1 | 0.25 | 0.12 | 32 | 4 | 4 | 8 | 2 | 16/8 |
| **SM2021061** | 4 | 0.5/9.5 | 4 | 0.25 | 0.12 | 16 | 16 | 0.25/4 | 16 | 0.25 | 0.25 | 32 | 4 | 4 | 32 | 32 | 32/16 |
| **SM2021063** | 4 | 0.5/9.5 | 8 | 0.25 | 0.12 | 0.25 | 0.25 | 0.25/4 | 16 | 0.25 | 0.25 | 32 | 4 | 4 | 32 | 32 | 32/16 |
| **SM2021073** | 32 | 8/152 | 8 | 0.25 | 0.12 | 16 | 16 | 0.25/4 | 16 | 0.25 | 2 | 32 | 8 | 4 | 32 | 32 | 32/16 |
| **SM2021074** | 32 | 8/152 | 8 | 0.25 | 0.12 | 16 | 4 | 0.25/4 | 16 | 0.5 | 2 | 32 | 4 | 4 | 32 | 32 | 32/16 |
| **SM2021076** | 32 | 8/152 | 8 | 0.25 | 0.12 | 16 | 16 | 0.25/4 | 16 | 0.25 | 2 | 32 | 64 | 64 | 32 | 32 | 32/16 |
| **SM2021077** | 32 | 8/152 | 8 | 0.25 | 0.12 | 16 | 16 | 0.25/4 | 16 | 0.25 | 2 | 32 | 32 | 64 | 32 | 32 | 32/16 |
| **SM2021080** | 32 | 8/152 | 8 | 0.25 | 0.12 | 16 | 8 | 0.25/4 | 16 | 0.25 | 2 | 32 | 4 | 64 | 32 | 32 | 32/16 |
| **SM2021186** | 4 | 0.5/9.5 | 8 | 0.25 | 0.12 | 0.25 | 0.25 | 0.25/4 | 1 | 0.25 | 0.015 | 32 | 4 | 4 | 8 | 2 | 2 |
| **SM2021187** | 4 | 0.5/9.5 | 4 | 0.25 | 0.12 | 0.25 | 0.25 | 0.25/4 | 16 | 0.25 | 0.25 | 32 | 4 | 4 | 32 | 16 | 16/8 |
| **SM2021189** | 4 | 0.5/9.5 | 4 | 0.25 | 0.12 | 0.25 | 0.25 | 0.25/4 | 16 | 0.25 | 0.25 | 32 | 4 | 4 | 32 | 32 | 32/16 |
| **SM2021210** | 8 | 8/152 | 4 | 0.25 | 0.12 | 16 | 16 | 0.25/4 | 16 | 0.25 | 0.25 | 32 | 32 | 4 | 32 | 32 | 32/16 |
| **SM2022003** | 4 | 0.5/9.5 | 4 | 0.25 | 0.12 | 0.25 | 0.5 | 0.25/4 | 16 | 0.25 | 0.25 | 32 | 4 | 4 | 32 | 32 | 2 |
| **SM2022077** | 4 | 1/19 | 4 | 0.25 | 0.12 | 0.25 | 0.5 | 0.25/4 | 1 | 0.25 | 0.12 | 32 | 4 | 4 | 16 | 32 | 16/8 |
| **SM2022087** | 4 | 0.5/9.5 | 4 | 0.25 | 0.12 | 0.25 | 0.5 | 0.25/4 | 1 | 0.25 | 0.25 | 32 | 4 | 4 | 32 | 32 | 32/16 |
| **SM2022110** | 4 | 0.5/9.5 | 8 | 0.25 | 0.12 | 0.25 | 0.25 | 0.25/4 | 1 | 0.25 | 0.25 | 32 | 4 | 4 | 8 | 2 | 2 |
| **SM2022112** | 4 | 1/19 | 4 | 0.25 | 0.12 | 0.25 | 0.25 | 0.25/4 | 2 | 0.25 | 0.12 | 32 | 2 | 4 | 8 | 32 | 32/16 |
| **SM2022122** | 4 | 1/19 | 4 | 0.25 | 0.12 | 0.25 | 0.25 | 0.25/4 | 2 | 0.25 | 0.25 | 32 | 4 | 4 | 16 | 32 | 16/8 |
| **SM2022142** | 4 | 0.5/9.5 | 4 | 0.25 | 0.12 | 0.25 | 0.25 | 0.25/4 | 16 | 0.25 | 0.25 | 32 | 4 | 4 | 32 | 32 | 32/16 |
| **SM2023002** | 4 | 0.5/9.5 | 4 | 0.25 | 0.12 | 0.25 | 0.25 | 0.25/4 | 16 | 0.5 | 0.25 | 32 | 4 | 4 | 32 | 32 | 16/8 |
| **SM2023003** | 4 | 0.5/9.5 | 8 | 0.25 | 0.12 | 0.25 | 0.25 | 0.25/4 | 16 | 0.5 | 0.25 | 32 | 4 | 4 | 32 | 32 | 32/16 |
| **SM2023028** | 4 | 2/38 | 4 | 0.25 | 0.12 | 0.25 | 0.5 | 0.25/4 | 1 | 0.5 | 0.12 | 8 | 2 | 4 | 16 | 32 | 16/8 |
| **SM2023031** | 4 | 0.5/9.5 | 8 | 0.25 | 0.12 | 0.25 | 0.5 | 0.25/4 | 1 | 0.5 | 0.25 | 32 | 4 | 4 | 32 | 32 | 32/16 |
| **SM2023040** | 4 | 0.5/9.5 | 4 | 0.25 | 0.12 | 0.25 | 8 | 0.25/4 | 16 | 0.5 | 0.25 | 32 | 4 | 4 | 32 | 32 | 32/16 |
| **SM2023055** | 4 | 0.5/9.5 | 4 | 0.25 | 0.12 | 2 | 8 | 0.25/4 | 16 | 0.5 | 0.25 | 32 | 4 | 4 | 32 | 32 | 32/16 |
| **SM2023129** | 4 | 8/152 | 4 | 0.25 | 0.12 | 16 | 16 | 0.25/4 | 16 | 0.25 | 0.25 | 32 | 8 | 4 | 32 | 32 | 32/16 |
| **SM2023157** | 32 | 8/152 | 4 | 0.25 | 0.12 | 0.25 | 0.5 | 0.5/4 | 16 | 0.25 | 0.25 | 32 | 4 | 4 | 32 | 32 | 32/16 |
| **SM2023167** | 32 | 8/152 | 4 | 0.25 | 0.12 | 0.25 | 8 | 0.5/4 | 16 | 0.5 | 0.5 | 32 | 4 | 4 | 32 | 32 | 32/16 |
| **SM2023180** | 4 | 0.5/9.5 | 4 | 0.25 | 0.12 | 0.25 | 0.25 | 0.25/4 | 1 | 0.25 | 0.25 | 32 | 4 | 4 | 32 | 32 | 32/16 |
| **SM2023181** | 4 | 0.5/9.5 | 4 | 0.25 | 0.12 | 0.25 | 0.25 | 0.25/4 | 1 | 0.25 | 0.12 | 32 | 4 | 4 | 32 | 32 | 16/8 |
| **SM2023193** | 4 | 0.5/9.5 | 4 | 0.25 | 0.12 | 0.25 | 0.25 | 0.25/4 | 1 | 0.5 | 0.25 | 32 | 4 | 4 | 32 | 32 | 32/16 |
| **SM2023252** | 4 | 0.5/9.5 | 4 | 0.25 | 0.12 | 0.25 | 0.25 | 0.25/4 | 1 | 0.25 | 0.25 | 32 | 4 | 4 | 32 | 32 | 32/16 |
| **SM2023277** | 4 | 0.5/9.5 | 4 | 0.25 | 0.12 | 0.25 | 0.5 | 0.25/4 | 2 | 0.25 | 0.25 | 32 | 4 | 4 | 32 | 32 | 32/16 |
| **SM2023280** | 8 | 0.5/9.5 | 4 | 0.25 | 0.12 | 0.25 | 0.5 | 0.25/4 | 2 | 0.25 | 0.25 | 32 | 4 | 4 | 32 | 32 | 32/16 |
| **resistance**  **(%)** | 10  (23.3) | 13  (30.2) | 43  (100) | 0  (0.0) | 0  (0.0) | 11  (25.6) | 8  (18.6) | 0  (0.0) | 24  (55.8) | 0  (0.0) | 6  (14.0) | 42  (97.7) | 4  (9.3) | 3  (7.0) | 33  (76.7) | 38  (88.4) | 30  (69.8) |
| Abbreviation: chloramphenicol (C), trimethoprim-sulfamethoxazole (SXT), colistin (CL), ertapenem (ETP), meropenem (MEM), cefotaxime (CTX), ceftazidime (CAZ), ceftazidime/avibactam (CZA), tetracycline (TE), tigecycline (TGC), ciprofloxacin (CIP), nalidixic acid (NA), azithromycin (AZM), amikacin (AN), streptomycin (STS), ampicillin (AM), ampicillin/sulbactam (SAM). | | | | | | | | | | | | | | | | | |

Supplementary Table S3. The characteristics of 43 CL-R *Salmonella* isolates based on WGS

| **Strain ID** | **Plasmid** | **Resistance pattern** | **Resistance genes** | **Mutation(gyrA)** |
| --- | --- | --- | --- | --- |
| SM2019009 | IncFIB, IncFII | CL+NA+STS | aac(6')-Iy, acrA,acrB, baeR, emrB, emrR, marA, CRP, golS, H-NS, sdiA | gyrA（D87G） |
| SM2019030 | IncFIB, IncFII, IncX1 | C+SXT+CL+CTX+TE+NA+AZM+STS+AM | bla_TEM-1_,aac(6')-Iy, tet(A), sul1,acrA, acrB, baeR, emrB, emrR, marA, CRP, golS, H-NS, sdiA | gyrA（D87G） |
| SM2019056 | IncFIB, IncFII, IncX1 | SXT+CL+TE+NA+AM+SAM | bla_TEM-1_,aac(6')-Iy, tet(A), sul1,acrA, acrB, baeR, emrB, emrR, marA, CRP, golS, H-NS, sdiA | gyrA（D87G） |
| SM2020121 | IncFIB, IncFII | C+SXT+CL+CTX+CAZ+TE+CIP+NA+STS+AM+SAM | bla_TEM-1_,aac(6')-Iy, aph(3'')-Ib, aph(6)-Id, tet(A), sul2, acrA, acrB, baeR, emrB, emrR, marA, CRP, golS, H-NS, sdiA | gyrA（D87Y） |
| SM2021002 | IncFIB, IncFII | CL+TE+NA+STS+AM+SAM | bla_TEM-1_,aac(6')-Iy, aph(3'')-Ib, aph(6)-Id, tet(A), sul2, acrA, acrB, baeR, emrB, emrR, marA, CRP, golS, H-NS, sdiA | gyrA（D87Y） |
| SM2021022 | IncFIB, IncFII | CL+NA+STS+AM+SAM | bla_TEM-1_,aac(6')-Iy, aph(3'')-Ib,aph(6)-Id, sul2, acrA, acrB, baeR, emrB, emrR, marA, CRP, golS, H-NS, sdiA | gyrA（D87Y） |
| SM2021032 | IncFIB, IncFII, IncX1 | CL+CTX+CAZ+NA+AM+SAM | bla_CTX-M-55_, bla_TEM-1_,aac(6')-Iy, aph(3')-IIa, acrA, acrB, baeR, emrB, emrR, marA, oqxA, CRP, golS, H-NS, sdiA | gyrA（D87Y） |
| SM2021041 | IncFIB, IncFII, IncX1 | C+SXT+CL+TE+NA+STS+AM+SAM | bla_TEM-1_,aac(6')-Iy, aadA5, acrB, emrB, emrR, marA, CRP, golS, H-NS, sdiA | — |
| SM2021044 | IncFIB, IncFII, IncX1 | CL+NA+AM | bla_TEM-1_,aac(6')-Iy, aadA5, acrA, acrB, baeR, emrB, emrR, marA, CRP, golS, H-NS, sdiA | gyrA（D87G） |
| SM2021045 | IncFIB, IncFII, IncX1 | CL+NA | bla_TEM-1_,aac(6')-Iy, aadA5, acrA, acrB, baeR, emrB, emrR, marA, CRP, golS, H-NS, sdiA | gyrA（D87G） |
| SM2021061 | IncI1-I | CL+CTX+CAZ+TE+NA+STS+AM+SAM | bla_CMY-2_, bla_TEM-1_,aac(6')-Iy, aph(3'')-Ib, aph(6)-Id, sul2, acrA, acrB, baeR, emrB, emrR, marA, CRP, golS, H-NS, sdiA | gyrA（D87Y） |
| SM2021063 | ColpVC, IncFIB, IncFII, IncI1-I | CL+TE+NA+STS+AM+SAM | bla_TEM-1_,aac(6')-Iy, aph(3'')-Ib, aph(6)-Id, tet(A), sul2, acrA, acrB, baeR, emrB, emrR, marA, CRP, golS, H-NS, sdiA | gyrA（D87Y） |
| SM2021073 | IncHI2, IncHI2A, IncQ1 | C+SXT+CL+CTX+CAZ+TE+CIP+NA+STS+AM+SAM | bla_CTX-M-14_, bla_OXA-1_, aph(3'')-Ib, aph(6)-Id,aac(6')-Iaa, aph(3')-Ia,aac(3)-Iva, aadA2, tet(B), tetR, sul1, sul3, mcr-1.1,aac(6')-Ib-cr6, qnrS2, acrA, acrB, baeR, emrB, emrR, marA, oqxA, CRP, golS, H-NS, sdiA | — |
| SM2021074 | p0111 | C+SXT+CL+CTX+TE+CIP+NA+STS+AM+SAM | bla_TEM-1_, APH(3'')-Ib, APH(6)-Id,aac(6')-Iaa, tet(A), sul2, qnrS1,acrA, acrB, baeR, emrB, emrR, marA, CRP, golS, H-NS, sdiA | — |
| SM2021076 | - | C+SXT+CL+CTX+CAZ+TE+CIP+NA+AZM+AN+STS+AM+SAM | bla_TEM-1_, aac(6')-Iaa, sul3, qnrS1, acrA, acrB, baeR, emrB, emrR, marA, CRP, golS, H-NS, sdiA | — |
| SM2021077 | IncFIB, IncFII, IncX1 | C+SXT+CL+CTX+CAZ+TE+CIP+NA+AZM+AN+STS+AM+SAM | bla_TEM-1_, aac(6')-Iy, aadA5, acrA, acrB, baeR, emrB, emrR, marA, CRP, golS, H-NS, sdiA | gyrA（D87G） |
| SM2021080 | IncFIB, IncFII, IncX1 | C+SXT+CL+CTX+TE+CIP+NA+AN+STS+AM+SAM | bla_TEM-1_,aac(6')-Iy, aadA5, acrA, acrB, baeR, emrB, emrR, marA, CRP, golS, H-NS, sdiA | gyrA（D87G） |
| SM2021186 | IncFIB, IncFII | CL+NA | aac(6')-Iy, acrA, acrB, baeR, emrB, emrR, marA, CRP, golS, H-NS, sdiA | gyrA（D87Y） |
| SM2021187 | IncFIB, IncFII | CL+TE+NA+STS | bla_TEM-1_,aac(6')-Iy, aph(3'')-Ib, aph(6)-Id, tet(A), sul2, acrA, acrB, baeR, emrB, emrR, marA, kdpE, CRP, golS, H-NS, sdiA | gyrA（D87Y） |
| SM2021189 | IncFIB, IncFII | CL+TE+NA+STS+AM+SAM | bla_TEM-1_,aac(6')-Iy, aph(3'')-Ib, aph(6)-Id, sul2, acrA, acrB, baeR, emrB, emrR, marA, kdpE, CRP, golS, H-NS, sdiA | gyrA（D87Y） |
| SM2021210 | IncFIB, IncFII | SXT+CL+CTX+CAZ+TE+NA+AZM+STS+AM+SAM | bla_TEM-1_,aac(6')-Iy, aph(3'')-Ib, aph(6)-Id, tet(A), sul2, acrA, acrB, baeR, emrB, emrR, marA, CRP, golS, H-NS, sdiA | gyrA（D87Y） |
| SM2022003 | IncFIB, IncFII | CL+TE+NA+STS+AM | bla_TEM-1_,aac(6')-Iy, aph(3'')-Ib, aph(6)-Id, tet(A), sul2, acrA, acrB, baeR, emrB, emrR, marA, CRP, golS, H-NS, sdiA | gyrA（D87Y） |
| SM2022077 | IncFIB, IncFII, IncX1 | CL+NA+AM | bla_TEM-1_,aac(6')-Iy, aadA5, acrA, acrB, baeR, emrB, emrR, marA, CRP, golS, H-NS, sdiA | gyrA（D87G） |
| SM2022087 | IncFIB, IncFII | CL+NA+STS+AM+SAM | bla_TEM-1_,aac(6')-Iy, aph(3'')-Ib, aph(6)-Id, sul2, acrA, acrB, baeR, emrB, emrR, marA, CRP, golS, H-NS, sdiA | gyrA（D87Y） |
| SM2022110 | IncFIB, IncFII | CL+NA | aac(6')-Iy, acrA, acrB, baeR, emrB, emrR, marA, CRP, golS, H-NS, sdiA | gyrA（D87Y） |
| SM2022112 | IncX1 | CL+NA+AM+SAM | bla_TEM-1_,aac(6')-Iy, aadA5, acrA, acrB, emrB, emrR, marA, CRP, golS, H-NS, sdiA | gyrA（D87G） |
| SM2022122 | IncFIB, IncFII, IncX1 | CL+NA+AM | bla_TEM-1_,aac(6')-Iy, aadA5, acrA, acrB, baeR, emrB, emrR, marA, CRP, golS, H-NS, sdiA | gyrA（D87G） |
| SM2022142 | IncFIB, IncFII | CL+TE+NA+STS+AM+SAM | bla_TEM-1_,aac(6')-Iy, aph(3'')-Ib, aph(6)-Id, tet(A), sul2, acrA, acrB, baeR, emrB, emrR, marA, CRP, golS, H-NS, sdiA | gyrA（D87Y） |
| SM2023002 | IncFIB, IncFII, IncX1 | CL+TE+NA+STS+AM | bla_TEM-1_,aac(6')-Iy, aadA5, acrA, acrB, baeR, emrB, emrR, marA, CRP, golS, H-NS, sdiA | gyrA（D87G） |
| SM2023003 | IncFIB, IncFII | CL+TE+NA+STS+AM+SAM | bla_TEM-1_,aac(6')-Iy, aph(3'')-Ib, aph(6)-Id, tet(A), sul2, acrA, acrB, baeR, emrB, emrR, marA, CRP, golS, H-NS, sdiA | gyrA（D87Y） |
| SM2023028 | IncFIB, IncFII, IncX1 | CL+AM | bla_TEM-1_,aac(6')-Iy, aadA5, acrA, acrB, baeR, emrB, emrR, marA, CRP, golS, H-NS, sdiA | gyrA（D87G） |
| SM2023031 | IncFIB, IncFII | CL+NA+STS+AM+SAM | bla_TEM-1_,aac(6')-Iy, aph(3'')-Ib, aph(6)-Id, tet(A), sul2, acrA, acrB, baeR, emrB, emrR, marA, CRP, golS, H-NS, sdiA | gyrA（D87Y） |
| SM2023040 | IncFIB, IncFII | CL+TE+NA+STS+AM+SAM | bla_TEM-1_,aac(6')-Iy, aph(3'')-Ib, aph(6)-Id, tet(A), sul2, acrA, acrB, baeR, emrB, emrR, marA, CRP, golS, H-NS, sdiA | gyrA（D87Y） |
| SM2023055 | IncFIB, IncFII | CL+TE+NA+STS+AM+SAM | bla_TEM-1_,aac(6')-Iy, aph(3'')-Ib, aph(6)-Id, tet(A), sul2, acrA, acrB, baeR, emrB, emrR, marA, CRP, golS, H-NS, sdiA | gyrA（D87Y） |
| SM2023129 | IncFIB, IncFII | SXT+CL+CTX+CAZ+TE+NA+STS+AM+SAM | bla_TEM-1_,aac(6')-Iy, aph(3'')-Ib, aph(6)-Id, sul2, acrA, acrB, baeR, emrB, emrR, marA, CRP, golS, H-NS, sdiA | gyrA（D87Y） |
| SM2023157 | IncFIB, IncFII | C+SXT+CL+TE+NA+STS+AM+SAM | bla_TEM-1_,aac(6')-Iy, aph(3'')-Ib, aph(6)-Id, sul2, acrA, acrB, baeR, emrB, emrR, marA, CRP, golS, H-NS, sdiA | gyrA（D87Y） |
| SM2023167 | IncFIB, IncFII | C+SXT+CL+TE+NA+STS+AM+SAM | bla_TEM-1_,aac(6')-Iy, aph(3'')-Ib, aph(6)-Id, sul2, acrA, acrB, baeR, emrB, emrR, marA, CRP, golS, H-NS, sdiA | gyrA（D87Y） |
| SM2023180 | IncFIB, IncFII | CL+NA+STS+AM+SAM | bla_TEM-1_,aac(6')-Iy, aph(3'')-Ib, aph(6)-Id, sul2, acrA, acrB, baeR, emrB, emrR, marA, CRP, golS, H-NS, sdiA | gyrA（D87Y） |
| SM2023181 | IncFIB, IncFII, IncX1 | CL+NA+STS+AM | bla_TEM-1_,aac(6')-Iy, aph(6)-Id, aadA5, acrA, acrB, baeR, emrB, emrR, marA, CRP, golS, H-NS, sdiA | gyrA（D87G） |
| SM2023193 | IncFIB, IncFII | CL+NA+STS+AM+SAM | bla_TEM-1_,aac(6')-Iy, aph(3'')-Ib, aph(6)-Id, sul2, acrA, acrB, baeR, emrB, emrR, marA, CRP, golS, H-NS, sdiA | gyrA（D87Y） |
| SM2023252 | IncFIB, IncFII | CL+NA+STS+AM+SAM | bla_TEM-1_,aac(6')-Iy, aph(3'')-Ib, aph(6)-Id, sul2, acrA, acrB, baeR, emrB, emrR, marA, CRP, golS, H-NS, sdiA | gyrA（D87Y） |
| SM2023277 | IncFIB, IncFII | CL+NA+STS+AM+SAM | bla_TEM-1_,aac(6')-Iy, aph(3'')-Ib, aph(6)-Id, sul2, acrA, acrB, baeR, emrB, emrR, marA, CRP, golS, H-NS, sdiA | gyrA（D87Y） |
| SM2023280 | IncFIB, IncFII | CL+NA+STS+AM+SAM | bla_TEM-1_,aac(6')-Iy, aph(3'')-Ib, aph(6)-Id, sul2, acrA, acrB, baeR, emrB, emrR, marA, CRP, golS, H-NS, sdiA | gyrA（D87Y） |

Supplementary Table S4. The details of 12 variations in 40 CL-R *S.* Enteritidis isolates.

| Gene | position | P value | variant | af |
| --- | --- | --- | --- | --- |
| SEN0278 | 319784 | 0.00539 | C_T | 0.444 |
| SEN0327 | 368418 | 0.00539 | C_A | 0.444 |
| SEN0707 | 785188 | 0.00539 | C_T | 0.444 |
| SEN0735 | 814636 | 0.00539 | C_T | 0.444 |
| dinG | 846624 | 0.00539 | A_G | 0.444 |
| focA | 973273 | 0.00539 | C_T | 0.444 |
| yccT | 1047507 | 0.00539 | C_T | 0.444 |
| pdxH | 1705949 | 0.00539 | G_A | 0.444 |
| ydiK | 1783261 | 0.00539 | G_A | 0.444 |
| SEN2191 | 2299657 | 0.00539 | A-G | 0.444 |
| ygcB | 2968763 | 0.00539 | G_A | 0.444 |
| SEN2929 | 3136576 | 0.00539 | A_C | 0.444 |
